# The Fifth Pair of Nerves

**Published:** 1869-11

**Authors:** William S. Carruthers


### ARTICLE III

### *The Fifth Pair of Nerves.*

By William S. Carruthers, D.D.S.

To the Dental practitioner an anatomical, physiological and pathological knowledge of the Trifacial is highly interesting, as well as essential, to an intelligent performance of his duties, for many of its painful affections are traceable to the derangement of the teeth, supplying, as it does, these organs with nerve filaments from the maxillary divisions.

Anatomically considered, we find the fifth nerve peculiar in being both compound and special, supplying parts with filaments of motion, with filaments of sensation, and through its gustatory branch with filaments pertaining to the sense of taste. It is the great sensitive nerve of the head and face, and from its vast peripheral expanse, and having its centre origin in the medulla oblongata, one of the most complex of nerve centres, explains the difficulty of diagnosing many of its affections.

The three divisions coming from the ganglion of Gasser are sensitive, the Opthalmic with its divisions, the frontal, lachrymal, and nasal branches, is distributed over the frontal orbito region, and their ramifications supply with filaments the muscles of the eyelid and forehead, integument of the forehead and nose, the tentorium, lachrymal gland, the pericranium of frontal, and parietal regions and ciliary muscle and iris. The supra-orbital branch of the frontal anastomoses with filaments of the facial nerve, and supplies the integument as far back as the occiput.

The second division of the Sup. Maxillary has the same origin, it is sensitive, and although smaller than the third division has a great range of distribution, associating with the facial nerve and supplying with sensor filaments, Meckel's ganglion. Its branches of distribution are, three from the spheno-maxillary fossa, the orbital, ganglionic and posterior dental, one from the infra orbital canal, and three on the face. The distribution and ramifications of this division are easily seen by the markings on the bones of the face, the orbital branch from the orbital cavity dividing into two branches the temporal and malar. The temporal passes through the foramen in the malar bone, enters the temporal fossa, and, associating with the facial and inferior maxillary, is distributed to the integument on the side of the head. The malar branch, also leaving the orbit through the foramen in the malar bone, perforates the orbicularis palpebrarum muscle, associating likewise with the facial nerve. The posterior dental dividing into two branches, the anterior and posterior, the latter entering the sup. maxillary bone above the tuberosity, forms a plexus and distributes filaments to the posterior teeth, the anterior supplying the gums and buccinator muscle, and their terminations lost in union with the other branch. The anterior dental branch we find coming from the infra-orbital canal, about midway entering another canal situated on the face of the maxillary sinus, this branch supplying the superior incisors, canines and bicuspid teeth. From the infra-orbital foramen we have the palpebral, labial and nasal filaments, supplying the obicularis palpebrarum muscle, and the integument and conjunctiva of the lower eye-lid, and associating at the outer angle of the orbit with the malar branch of the orbital, and filaments of the facial nerve, the nasal filaments supplying the muscles and integuments of the nose and this region, and the labial filaments distributed to the muscles and integument of the upper lip.

The inferior maxillary nerve is the largest of the three divisions, and is compound, being sensor and motor, the sensor coming from the ganglia and uniting with the motor cord just beneath. There are two branches of this division, anterior and posterior, the anterior by five divisions supplies the masseter muscle, the deep surface of temporal and the buccinator and its integument and mucous membrane. The posterior branch, the larger of the two divisions, is subdivided into three branches, the inferior dental, the lingual and auriculo-temporal, and supplies the inferior teeth, the tongue and auriculo-temporal region. The lingual is the nerve of special sense, and is distributed to the anterior two-thirds of the tongue, its terminal filament to the papillæ, the filaform and fungiform, the posterior being supplied from the glosso-pharyngeal.

The inferior dental branch is the largest of the three divisions, and after giving a branch to the mylo-hyoid and to the anterior belly of the digastric, it enters the posterior foramen of the dental canal and passing beneath the teeth, gives in its course filaments to these organs, terminating in the mental branch, which emerges from the canal at the mental foramen and distributes filaments to the muscles and skin of the inferior lip.

The function of the fifth nerve has been determined from repeated experiments, and from the analogy it bears to the spinal nerves, thus by tracing each of its three great divisions we may determine its function by its constitution, according as it derives its fibres from either root, or from both, and the distribution of these divisions confirm the views of its physiology suggested by the anatomy of its origin. Division of the opthalmic, or of the superior maxillary, causes loss of sensibility without muscular paralysis, but with the incision of the inferior maxillary then the power of mastication is destroyed and the sensibility of the lower part of the face and tongue. The fifth nerve may therefore be regarded as the motor nerve of mastication, and the sensitive nerve to all of the surface external and internal which belongs to the face and anterior part of the cranium.

We have thus arrived at a correct knowledge of the anatomical and physiological connection and the results of physical changes, yet there are many painful affections which constantly embarras us, that may be traced to the derangement of the teeth (these organs being more subject to disease and abnormal conditions, and cause as much constitutional irritation as any other organs of the various systems of the animal economy), and which have been considered by the medical profession (who generally know very little of dental pathology) as neuralgic, rheumatic, idiopathic, &c. To dental students the honor is reserved of elucidating them, as was reserved the honor of introducing anæsthetics for surgical operations, and thus further illustrating the advantage and necessity of a knowledge of the various branches (as taught in our Dental Colleges) to the dental art.

Many of the painful affections of this nerve are due to reflex action, and it is interesting to us, as being the great channel of reflex impressions between various important nerves already mentioned, and with which it possesses intimate relations, or rather connections, either at its central origin in its courses, or at its peripheral termination. And the sensory division with the motor root is perhaps one of the most important connections in a practical view with facial neuralgic affections.

It not unfrequently happens that parts remote become the seat of pain from the exposure of the nerve of a tooth, as amaurosis and deafness. Dr. Tilbnry Fox records a case of moral insanity cured by the removal of some decayed teeth. In the dentition of children, whether primary or secondary, it is always more or less affected, and the irritation of it, caused by the pressure of the teeth, often bring about convulsions, the brain and spinal cord being affected. The foundation of nervous disorders of subsequent years may be laid in this manner at the period referred to. We might also enumerate many other disorders produced by the influence of first dentition, from cutaneous eruption to lockjaw. In facial neuralgic affections when this nerve, together with the facial, becomes involved by disease, it is capable of producing the greatest amount of physical suffering of any of the nerves of the body, and, as through its normal healthy condition the tastes and gratifications of the will, the imaginations of the mind, and the necessities and luxuries of life are enjoyed, so on the other hand disease will produce equally as great misery as its healthy condition is conducive to happiness.

